# Mast-cell expressed membrane protein-1 is expressed in classical monocytes and alveolar macrophages in idiopathic pulmonary fibrosis and regulates cell chemotaxis, adhesion, and migration in a TGFβ-dependent manner

**DOI:** 10.1152/ajpcell.00563.2023

**Published:** 2024-01-08

**Authors:** Carole Y. Perrot, Theodoros Karampitsakos, Avraham Unterman, Taylor Adams, Krystin Marlin, Alyssa Arsenault, Amy Zhao, Naftali Kaminski, Gundars Katlaps, Kapilkumar Patel, Debabrata Bandyopadhyay, Jose D. Herazo-Maya

**Affiliations:** ^1^Division of Pulmonary, Critical Care and Sleep Medicine, Department of Internal Medicine, Ubben Center for Pulmonary Fibrosis Research, Morsani College of Medicine, University of South Florida, Tampa, Florida, United States; ^2^Section of Pulmonary, Critical Care and Sleep Medicine, Department of Internal Medicine, School of Medicine, Yale University, New Haven, Connecticut, United States; ^3^Pulmonary Fibrosis Center of Excellence, Tel Aviv Sourasky Medical Center, Sackler School of Medicine, Institute of Pulmonary Medicine, Tel Aviv University, Tel Aviv, Israel; ^4^Genomic Research Laboratory for Lung Fibrosis, Tel Aviv Sourasky Medical Center, Tel Aviv University, Tel Aviv, Israel; ^5^Division of Cardiothoracic Surgery, Department of Surgery, Morsani College of Medicine, University of South Florida, Tampa, Florida, United States; ^6^Center for Advanced Lung Disease and Lung Transplant Program, Tampa General Hospital, Tampa, Florida, United States

**Keywords:** alveolar macrophages, idiopathic pulmonary fibrosis, mast-cell expressed membrane protein-1, monocytes

## Abstract

Mast-cell expressed membrane protein-1 (MCEMP1) is higher in patients with idiopathic pulmonary fibrosis (IPF) with an increased risk of death. Here we aimed to establish the mechanistic role of MCEMP1 in pulmonary fibrosis. We identified increased MCEMP1 expression in classical monocytes and alveolar macrophages in IPF compared with controls. MCEMP1 is upregulated by transforming growth factor beta (TGFβ) at the mRNA and protein levels in monocytic leukemia THP-1 cells. TGFβ-mediated MCEMP1 upregulation results from the cooperation of SMAD3 and SP1 via concomitant binding to SMAD3/SP1 *cis-*regulatory elements within the MCEMP1 promoter. We also found that MCEMP1 regulates TGFβ-mediated monocyte chemotaxis, adhesion, and migration. Our results suggest that MCEMP1 may regulate the migration and transition of monocytes to monocyte-derived alveolar macrophages during pulmonary fibrosis development and progression.

**NEW & NOTEWORTHY** MCEMP1 is highly expressed in circulating classical monocytes and alveolar macrophages in IPF, is regulated by TGFβ, and participates in the chemotaxis, adhesion, and migration of circulating monocytes by modulating the effect of TGFβ in RHO activity.

## Graphical abstract

**Figure:**
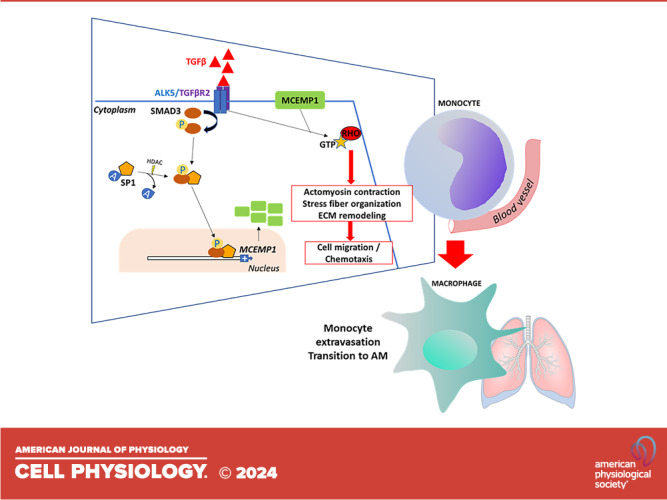

## INTRODUCTION

Idiopathic pulmonary fibrosis (IPF) is a chronic and progressive interstitial lung disease (ILD) characterized by repetitive epithelial cell injury, aberrant deposition of extracellular matrix, immune deregulation, irreversible lung tissue scarring, and subsequently impaired lung function (1, 2). Current antifibrotic therapies are only able to slow lung function decline (3, 4). Thus, disease progression is inevitable. However, the pattern of disease progression is highly heterogeneous, with some patients demonstrating long-term clinical stability and others experiencing a more rapid disease course (5–7). This fueled extensive research efforts for the identification of biomarkers predictive of IPF progression and mortality (5).

We previously used genome-wide, transcript profiling of peripheral blood mononuclear cells to identify expression patterns predictive of IPF survival and validated a peripheral blood 52-gene signature predictive of transplant-free survival and mortality in patients with IPF from six different cohorts (8, 9). We have previously shown by cellular deconvolution and single-cell RNA sequencing that classical monocytes in peripheral blood are the source of increased gene expressions predictive of mortality in IPF (10). Mast-cell expressed membrane protein-1 (*MCEMP1*, c19orf59) a novel and poorly characterized gene, was among the upregulated genes of the 52-gene signature (9). The *MCEMP1* gene encodes a type II transmembrane protein, and its expression has been identified in monocytic leukemia cell lines (THP-1) and in lung mast cells (11).

Although we studied peripheral blood gene expression changes predictive of IPF mortality, we did not study the cellular source and the mechanisms of *MCEMP1* in the pathogenesis of pulmonary fibrosis. In this study, we showed that *MCEMP1* is expressed in circulating monocytes and in alveolar macrophages in IPF. We also identified that *MCEMP1* is transcriptionally regulated by transforming growth factor beta (TGFβ) in a SMAD3/SP1-dependent manner. Our study shows that *MCEMP1* might modulate TGFβ-mediated monocyte adhesion, chemotaxis, and migration by regulating RHO-GTPase activity. Our results demonstrate that *MCEMP1* is a TGFβ target gene that may be critical in the migration and transition of circulating monocytes to monocyte-derived alveolar macrophages during lung injury and aberrant repair.

## MATERIAL AND METHODS

### Single-Cell RNA Sequencing Data Analysis

We reanalyzed peripheral blood mononuclear cell single-cell RNA sequencing (scRNA-Seq) data from *n* = 25 patients with IPF and *n* = 13 controls recruited at Yale University as previously reported (12). Comparison of *MCEMP1* expression levels between monocytes of patients with IPF and controls was conducted using the Wilcoxon rank sum test. We also reanalyzed single-cell RNA sequencing data set including 312,928 cells from distal lung parenchyma samples obtained from 32 IPF lungs (45 libraries yielding 147,169 cells), 18 chronic obstructive pulmonary disease (COPD) lungs (24 libraries yielding 69,456 cells), and 28 control donor lungs (38 libraries, yielding 96,303 cells) (13).

### Detection of CD206, MCEMP1, and HT2-280 in Human Lungs by Immunofluorescence Staining and Quantification

Normal lung tissue slides were purchased from Origene (Rockville, MD) and were used as controls. IPF lung tissues were explants derived from three patients undergoing lung transplantation at Tampa General Hospital. Briefly, fresh human lung tissue was collected, placed on ice-chilled Petri dishes, and cut into 5-mm thick pieces. Lung tissues were washed twice in 1X PBS, fixed in 10% formalin overnight at 4°C on a rocker, progressively dehydrated in 70%, 80%, 90%, 95% and 100% ethanol, cleared in xylene (2 times for 30 min) and embedded in paraffin (3 times for 60 min) using a Leica Automatic Tissue Processor (Wetzlar, Germany). Paraffin tissue blocks were left overnight at room temperature to solidify before sectioning. Sample collection was approved by the local Institutional Review Board (IRB) board. For immunofluorescence staining, tissue sections were deparaffinized, hydrated, immersed in a pH = 6 antigen retrieval solution (IHC World, Elliott City, MD) and placed in a steamer for 40 min at 95°C–98°C. Slides were allowed to cool down for 20 min, then rinsed with water and air-dried completely.

Tissue sections were incubated in blocking buffer (Triton X-100 0.1%, goat serum 10%, BSA 3%, 1X PBS) for an hour at room temperature, followed by overnight incubation with different combinations of primary antibodies targeting CD206 (5 µg/mL), MCEMP1 (1.5 µg/mL), and type-2 alveolar epithelial cell marker HT2-280 (1/200) at 4°C. Tissue sections were washed three times with 1X PBS and incubated with combinations of Alexa-Fluor 488, 555, and 647 secondary antibodies (1/1,000 dilution; Thermo Fisher Scientific) for 1 h at room temperature followed by DAPI for 5 min. Tissue sections were washed three times with 1X PBS, mounted under a coverslip using Cytoseal 60, and stored at 4°C overnight before image capture using a Nikon Eclipse Ni-E fluorescence microscope and NIS-Elements software.

MCEMP1 and CD206 staining were quantified using NIS Elements AR5-30-05 software. Macrophages identified as CD206^+^ cells, and MCEMP1^+^ cells were quantified by measuring the ratio CD206^+^ area/total tissue area and MCEMP1^+^ area/total tissue area, respectively, in >20 micrographs from lungs of two healthy individuals and three patients with IPF. MCEMP1^+^ macrophages were quantified by measuring MCEMP1^+^; CD206^+^ cells/total CD206^+^ cells. The proportion of macrophages among MCEMP1^+^ cells was measured with the ratio MCEMP1^+^; CD206^+^ cells/total MCEMP1^+^ cells. Statistical analysis was performed using one-way ANOVA with Sidak post hoc test for multiple comparisons.

### Cell Culture and Reagents

THP-1 and U937 cell lines (Millipore Sigma, Burlington, MA) were cultured in RPMI 1640 medium with l-glutamine (Gibco, Thermo Fisher Scientific, Waltham, MA) supplemented with 10% fetal bovine serum and antibiotics. All our experiments were performed using THP-1 and U937 at *passage <25*. Recombinant human TGFβ1, FGF2, TNF-α, IL14, IL10, and IL14 (Peprotech, Cranbury, NJ) and LPS (Thermo Fisher Scientific) were used to stimulate THP-1 cells. Phorbol 12-myristate 13-acetate (PMA) was used for monocyte-to-macrophage differentiation. SMAD3 inhibitor SiS3 and HDAC inhibitor trichostatin A were purchased from Millipore Sigma and Mithramycin A from Cayman Chemical (Ann Harbor, MI).

### Antibodies

For Western blot and immunostaining applications, we used a polyclonal rabbit anti-MCEMP1 antibody from Millipore Sigma (ref. HPA014731). For Western blot only, monoclonal rabbit anti-SMAD3 (ref. 9523), anti-phospho-SMAD3 (ref. 9520), anti-SMAD2 (ref. 5339), anti-phospho-SMAD2 (ref. 18338), and anti-GAPDH (ref. 5174) antibodies were purchased from Cell Signaling Technology (Danvers, MA). All secondary HRP-conjugated antibodies were obtained from Promega (Madison, WI). For immunofluorescence only, monoclonal mouse anti-CD206 (MRC1; ref. Amab90746) was from Millipore Sigma, monoclonal mouse anti-CD14 (ref. MA-1–23611) was from Invitrogen (Thermo Fisher Scientific), and monoclonal mouse anti-HT2-280 (ref. T-27) was from Terrace Biotech (San Francisco, CA). Alexa Fluor 647 Phalloidin as well as Alexa Fluor secondary antibodies (488, 555, and 647) were purchased from Thermo Fisher Scientific. For chromatin immunoprecipitation, we used ChIP-grade monoclonal rabbit anti-SP1 (ref. ab231778) and anti-SMAD3 (ref. ab208182) antibodies from Abcam (Cambridge, MA). For proximity ligation assay, we used the anti-SP1 antibody mentioned earlier and a monoclonal mouse anti-SMAD3 antibody from Proteintech (Rosemont, IL).

### RNA Extraction and RT-qPCR

RNA extraction was conducted using the Rneasy Plus kit from Qiagen (Hilden, Germany) following the manufacturer’s instructions. Between 500 ng and 1 µg of RNA was then subjected to DnaseI, Amplification Grade digestion (Thermo Fisher Scientific), followed by reverse transcription using Superscript IV First-Strand Synthesis System (Thermo Fisher Scientific). Quantitative polymerase chain reaction (qPCR) was then performed using Power SYBR Green PCR Master Mix (Thermo Fisher Scientific) on a CFX384 Real-Time PCR detection system (Bio-Rad, Hercules, CA). Primer sets for *MCEMP1*, *RPS18*, and *RPL37A* were designed using Primer Blast (14) and empirically validated for quantitative PCR use following primer efficiency calculation. All our qPCR experiments were repeated at least three times, and samples were run in triplicates.

### Protein Isolation and Western Blot

Total cell proteins were extracted using radioimmunoprecipitation (RIPA) buffer supplemented with protease inhibitor cocktail and phosphatase inhibitors, and bicinchoninic acid (BCA) assay was performed to determine protein concentration (Thermo Fisher Scientific). Western blotting was performed by electrophoresis of 20 µg of proteins on Mini-PROTEAN or Criterion TGX Precast Gels (Bio-Rad, Hercules, CA), followed by electrotransfer to Amersham Protran nitrocellulose membrane (Millipore Sigma). After blocking unspecific binding, antibody incubations were carried out overnight in blocking buffer (5% BSA or 5% nonfat milk in TBS containing 0.1% Tween-20), and target proteins were detected using Western Lightning Plus-ECL (PerkinElmer, Waltham, MA) and a Chemidoc imaging system (Bio-Rad). Blots were quantified using ImageJ. All our WB experiments were repeated at least three times, and samples were pooled from three different culture vessels.

### Live Cell Imaging

THP-1 cells were seeded in six-well plates and treated with TGFβ (5 ng/mL) for 48 h, followed by an overnight incubation with PMA (150 nM). Cells were washed three times with 1X PBS and incubated with MCEMP1 (1/200) or CD14 (1/200) antibodies diluted in ice-cold assay buffer (1X PBS, BSA 2%, NaN_3_ 0.5%) for 2 h at 4°C. After two more washes with the assay buffer, cells were incubated with Alexa Fluor 555 secondary antibodies for 1.5 h at 4°C, washed twice with the assay buffer, and resuspended in PBS for observation and picture acquisition using a Nikon fluorescence microscope.

### Chromatin Immunoprecipitation

THP-1 cells were grown in T75 flasks for 24 h, then treated with SiS3 (25 µM), mithramycin A (100 nM), trichostatin A (10 µM), or vehicle for 1 h before TGFβ treatment. Twenty-four hours later, cells were harvested, spun down at 300 *g* for 5 min, and cell pellets were resuspended in 1% formaldehyde fixation solution for 12 min at room temperature under gentle agitation. Chromatin immunoprecipitation (ChIP) was carried out using the ChIP-IT Express Enzymatic kit from Active Motif (Rixensart, Belgium). In brief, 10 μg of enzymatically sheared chromatin was incubated overnight at 4°C using 3 μg of either control anti-IgG, ChIP grade anti-SMAD3 (Abcam), or ChIP grade anti-SP1 (Abcam) antibodies along with protein G magnetic beads. Precipitated chromatin was eluted, cross-links reversed, submitted to proteinase K treatment, and processed for quantitative PCR analysis of SMAD3 and SP1 binding to the human MCEMP1 promoter using Power SYBR Green PCR Master Mix (Thermo Fisher Scientific). Chromatin immunoprecipitation was performed using cells pooled from three different T75 culture flasks (a total of ∼45–60 million cells) treated independently, for each condition. qPCR was performed in three technical replicates, and results were calculated using the ΔΔCt method and are shown as percentage of input DNA for each ChIP experiment.

### Proximity Ligation Assay

The DuoLink in Situ Detection reagents Orange (λex = 554 nm and λem = 576 nm; Millipore Sigma) was used to determine whether SMAD3 interacts with SP1 in THP-1 cells. Briefly, PMA-treated cells were grown in four-well chamber slides until attachment, then subjected to SiS3, mithramycin A, or vehicle treatment followed by TGFβ for 24 h. Cells were washed with 1× PBS, fixed with a 4% paraformaldehyde solution for 10 min, permeabilized for 40 min at room temperature, blocked with the Duolink Blocking Solution for 1 h at 37°C, and incubated overnight at 4°C with mouse anti-SMAD3 (Proteintech, 1:400) and rabbit anti-SP1 (Abcam, 1:200) antibodies. Samples were then incubated with DNA-conjugated secondary antibodies (anti-rabbit probe PLUS and anti-mouse probe MINUS, 1:5 dilution) at 37°C for 1 h. Our negative control consisted of a sample incubated with secondary antibodies only. Hybridization, ligation, amplification, and detection steps were performed according to the manufacturer’s instructions. Coverslips were mounted on slides with mounting medium with 4′,6-diamidino-2-phenylindole (DAPI) from the kit and kept at −20°C overnight before image capture and analysis using a Nikon Eclipse Ni-E fluorescence microscope and NIS-Elements software. Proximity ligation assay was performed in three independent culture dishes, and at least five pictures were taken in each vessel for signal quantification.

### Transient RNA Interference

For *MCEMP1* silencing, THP-1 cells were transfected at 600,000 cells/mL in RPMI 10% FBS with 10 nM Silencer Select siRNA (Ambion; sense 5′GAAUGUCUCAAACUCCGUAtt— antisense 5′UACGGAGUUUGAGACAUUCca) using Lipofectamine RNAiMAX Reagent (Thermo Fisher Scientific). Cells were collected 48- and 72-h posttransfection for analyses.

### Lentivirus Transduction

For *MCEMP1* stable silencing and overexpression, THP-1 cells were transduced by spinofection with either pGFP-C-sh*MCEMP1* or -shctrl lentiviral vectors for silencing, and either with a lentiviral expression vector carrying GFP-tagged *MCEMP1* ORF or insertless (mock) vector for *MCEMP1* overexpression (lentivirus were purchased from Origene, MD). A MOI of 50 was necessary to achieve a satisfactory infection rate. Transduced cells were selected with puromycin (0.5 μg/mL) for 10 to 15 days and tested for *MCEMP1* expression by Western blot before use. All primer sets used for our study can be seen in Supplemental Table S1.

### Chemotaxis Assay

The Chemotaxis assay was performed using the Cell Migration/Chemotaxis Assay kit (24-well, 5 μm) from Abcam following the manufacturer’s instructions. Briefly, we performed transfection of THP-1 cells with control- or *MCEMP1*-targeting siRNA to knockdown *MCEMP1* expression, followed by TGFβ stimulation for 24 h. We also stably transduced THP-1 cells with mock—and *MCEMP1*—lentiviral vectors to induce *MCEMP1* expression. Cells from these experimental conditions were collected, washed with 1X PBS, resuspended in basal RPMI, and 200,000 cells were seeded into 5-µm pore-sized inserts of 24-well transwell plates. Serum-free medium (0.1% FBS) containing chemoattractant CCL2 (50 ng/mL) was dispensed in the bottom chambers of the plates, and cells were incubated at 37°C for 18 to 24 h. Inserts were carefully aspired and removed, and the plates were centrifuged at 1,000 *g* for 5 min. The number of migrated cells was determined by measuring the fluorescence signal intensity for each sample using a standard curve.

### Cell Adhesion Assay

THP-1 cells transfected either with control- or *MCEMP1*-targeting siRNA were cultured in six-well plates and stimulated with TGFβ (5 ng/mL) 24 h posttransfection. Cells were collected 24 h later, washed with 1X PBS, resuspended in serum-free medium, and seeded in 24-well plates (200,000 cells/well). After incubation for 1 h at 37°C, unattached cells were removed with PBS, and adherent cells were fixed with a 20% methanol/0.5% crystal violet solution for 15 min at room temperature. PMA-treated cells were used as positive control. Crystal violet was extracted from cells using 100 μL of ethanol 70%, and absorbance was measured at 595 nm. The assay was repeated three times in tri-or quadruplicates for each condition.

### Wound Closure (Scratch) Assay

Mock- and *MCEMP1*-overexpressing THP-1 cells (from 2 independent transduction experiments) were seeded in six-well plates at the density of 1 × 10^6^ cells/dish and immediately treated with 150 nM PMA to induce THP-1 differentiation into macrophages and cell adhesion. Forty-eight hours posttreatment, a scratch was performed with a 200 μL-pipette tip to create a wound in the confluent cell monolayer. Wound closure was monitored by taking bright-field pictures of the cells immediately after scratch and 72 h later using optical microscopy. The ImageJ software was used to measure wound closure areas. The experiment was performed in two independent wells for each condition, and our results represent the analysis of 10 micrographs.

### Bulk RNA Sequencing and Analysis

To study the mechanisms by which MCEMP1 participates in monocyte chemotaxis, we performed bulk RNA-sequencing using three batches of transduced cells (see *Lentivirus Transduction* section) that were subcultured and treated with TGFβ (5 ng/mL) or vehicle for 24 h before RNA extraction. RNA-seq was performed by Genewiz-Azenta Life Sciences (South Plainfield, NJ). Differential gene expression analysis was performed using the Deseq2 package. Three groups were analyzed: nontreated versus TGFβ-treated THP-1 cells, mock versus *MCEMP1* overexpressing cells treated with TGFβ and sh control versus sh*MCEMP1* treated with TGFβ. Differentially expressed genes (DEG) with *P* value <0.05 (FDR adjusted) were selected for downstream analyses. The complete datasets are available in the Gene Expression Omnibus database (http://www.ncbi.nlm.nih.gov/geo/) under accession number GSE247270.

### Active RHO Pull-Down Assay

THP-1 cells were stably transduced with control or MCEMP1-targeting shRNA using lentivirus and cultured in 75 cm^2^ flasks to reach at least 10 million cells per flask. Cells were further cultured with or without TGFβ (5 ng/mL) for 24 h, and pelleted by centrifugation (100 *g*, 5 min). After resuspension in ice-cold TBS, cells were centrifuged, lysed, and the supernatant was collected to measure protein concentration. For each sample, 1 mg of protein from a pool of three independent cell lysates for each condition was used to pull down active RHO (RHO-GTP) using the Active RHO Pull-Down and Detection kit from Thermo Fisher Scientific by following the manufacturer’s instructions. A volume of each cell lysate was used for detection of total RHO, MCEMP1, and GAPDH by Western blot.

### Statistical Analysis

Statistics were performed using GraphPad Prism 10. The two-tailed Student’s *t* test was used to compare two conditions. For multiple comparisons, we used either the one-way or two-way ANOVA with Dunnett’s, Tukey’s, or Sidak’s multiple-comparison test as appropriate for each comparison. Error bars represent standard deviation (SD). Graphic definition of statistical significance: *P* value <0.0001, *P* value <0.001, *P* value <0.01, and *P* value <0.05.

### Statistical Analysis of Bulk RNA Sequencing Data

Differential gene expression analysis was performed using Deseq2 package (15). Three groups were analyzed: nontreated versus TGFβ-treated cells, *MCEMP1* mock versus *MCEMP1* overexpressing cells treated with TGFβ and sh control versus sh*MCEMP1* treated with TGFβ. Differentially expressed genes (DEG) with *P* value <0.05 (FDR adjusted) were selected for downstream analyses. Gene Ontology (GO) analysis was performed on Bulk RNA sequencing. Statistical significance for GO analysis was defined as Bonferroni corrected *P* value <0.05.

## RESULTS

### *MCEMP1* Is Highly Expressed in Circulating Classical Monocytes and Alveolar Macrophages in IPF When Compared with Controls

**Figure 1.**
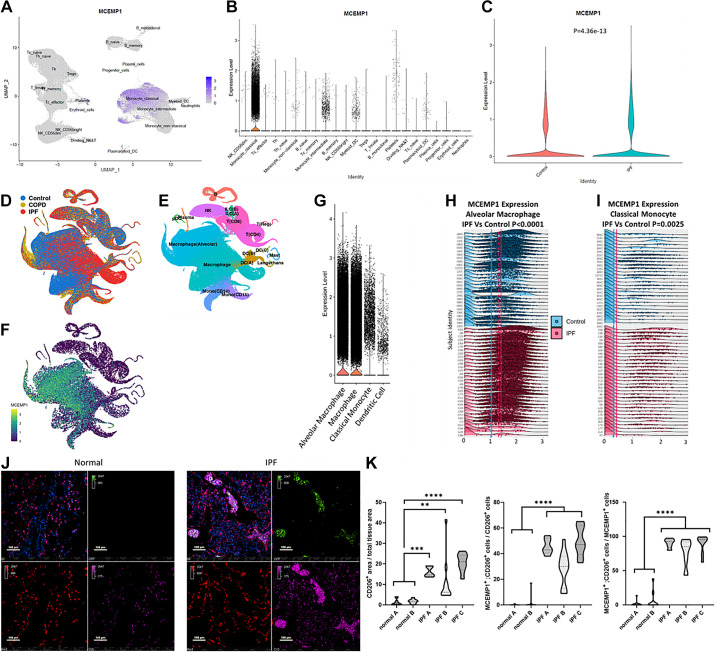
*MCEMP1* expression is increased in circulating classical monocytes and alveolar macrophages in patients with idiopathic pulmonary fibrosis (IPF) compared with controls. Uniform Manifold Approximation and Projection (UMAP) representation of 149,564 cells parceled into 23 cell types. All expected cell types are included. *MCEMP1* expression is colored purple, and color scale is adjacent to the UMAP. *MCEMP1* expression is highest in classical monocytes in IPF (*A*). Other cell types expressing *MCEMP1* to a lesser extent include intermediate monocytes, platelets, myeloid-derived, and plasmacytoid dendritic cells (*B*). Violin plots demonstrate that *MCEMP1* expression in classical monocytes is significantly increased in IPF (green) vs. controls (red) (*C*). Wilcoxon rank sum test, *P* < 0.0001. UMAP projections of RNA sequencing of 312,928 single cells from control, chronic obstructive pulmonary disease (COPD), and IPF lung tissues (*D*). Density plots show cell population density based on cellular markers (*E*) and *MCEMP1* expression (*F*). High expression of *MCEMP1* is detected in alveolar macrophages, macrophages, CD14^+^ monocytes, and dendritic cells (*G*). *MCEMP1* expression is significantly higher in alveolar macrophages (*H*) and in classical monocytes (*I*) in patients with IPF when compared with controls. *Y*-axis (subject identity), *x*-axis (expression levels). Immunofluorescence staining demonstrates absence of MCEMP1 staining in normal lungs and its presence and colocalization in alveolar macrophages in IPF (*J*). The difference between cells coexpressing CD206 and MCEMP1 was significantly higher in IPF compared with controls (*K*). DAPI: Blue, MCEMP1: Green, HT2-280: Red, and CD206: Purple. ****P < 0.0001, ***P < 0.001, **P < 0.01. MCEMP1, mast-cell expressed membrane protein-1.

To understand the cellular source of *MCEMP1* expression in IPF and whether its expression was different from age- and gender-matched healthy controls, we analyzed scRNA-Seq of peripheral blood mononuclear cells (PBMCs) from patients with IPF and controls (12). Table 1 summarizes the demographic differences of the studied subjects. Our analysis indicated that classical monocytes are the main cellular source of *MCEMP1* in PBMC (Fig. 1*A*). Although *MCEMP1* expression is higher in CD14^+^;CD16^−^ classical monocytes, it is also detected (albeit modestly) in CD14^+^;CD16^+^ intermediate monocytes, dendritic cells, and thrombocytes; however, *MCEMP1* is not expressed in CD14^−^; CD16^+^ nonclassical monocytes and in lymphocytes in IPF (Fig. 1*B*). In circulating classical monocytes, *MCEMP1* expression was significantly higher in patients with IPF compared with control subjects (Fig. 1*C*).

**Table 1.** Baseline characteristics of patients with IPF and control subjects in the PBMC scRNA-Seq dataset

	IPF	Controls	*P* Value
*n*	25	13	
Age means (SD), yr	70.3 (6.4)	71.0 (4.2)	0.70
Male sex	80%	77%	0.83
Caucasian race	96%	100%	0.46
Current/former smoker	72%	69%	0.86
FVC, % predicted	73.48	NA	NA
DLCO, % predicted	41.12	NA	NA

DLCO, diffusion capacity of the lungs for carbon monoxide; FVC, forced vital capacity; IPF, idiopathic pulmonary fibrosis; PBMC, peripheral blood mononuclear cell.

To determine the cellular source of *MCEMP1* in IPF lung tissues, we analyzed scRNA-Seq cells of the distal lung parenchyma from 32 IPF lungs, 18 chronic obstructive pulmonary disease (COPD), and 28 control donor lungs (13). We identified that *MCEMP1* is primarily expressed in lung tissue in alveolar macrophages, macrophages, classical monocytes (CD14^+^), and dendritic cells (Fig. 1, *D*–*G*). *MCEMP1* expression was significantly higher in alveolar macrophages (Fig. 1*H*) and classical monocytes (Fig. 1*I*) in IPF when compared with control lungs. To validate our findings, we performed immunofluorescence staining and colocalization of MCEMP1 with CD206 in IPF lung tissue and control lungs. MCEMP1 staining was essentially absent in control lungs while it was detected and colocalized with CD206 in alveolar macrophages in IPF (Fig. 1*J*). The differences in the total number of CD206^ + ^MCEMP1^+^ cells between IPF and controls by immunofluorescence staining were statistically significant (Fig. 1*K*).

### *MCEMP1* Is a TGFβ Inducible Gene in Monocytes

**Figure 2.**
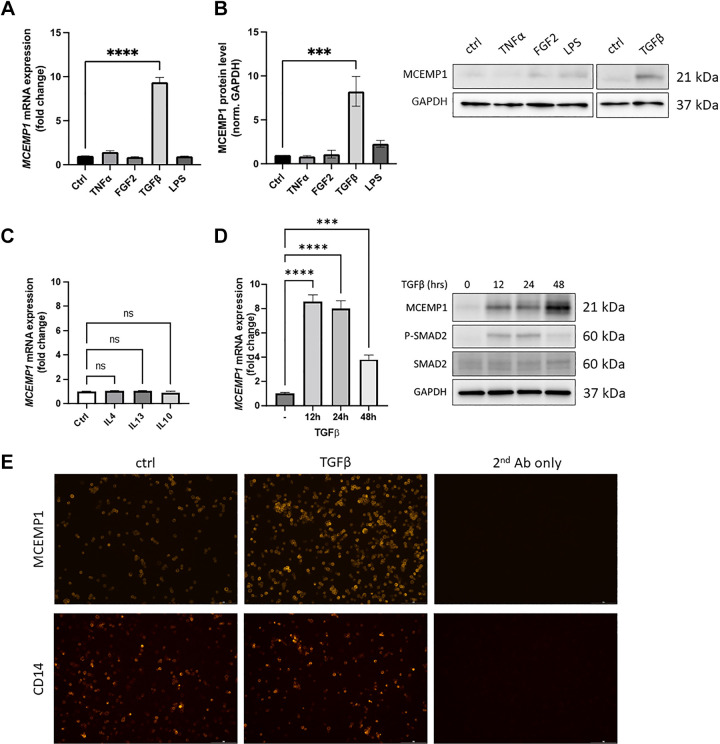
*MCEMP1* is a TGFβ inducible gene in monocytes. MCEMP1 was found to be upregulated by TGFβ at the gene expression (*A*) and protein levels (*B*). Stimulating THP-1 cells with fibroblast growth factor 2 (FGF2), tumor necrosis factor alpha (TNF-α), and lipopolysaccharide (LPS) did not significantly change MCEMP1 RNA and protein levels (*B*). Interleukins 4, 13, and 10 did not affect *MCEMP1* expression levels (*C*). The highest *MCEMP1* gene expression was observed at 12 h (*D*), Phospho-SMAD2 and total SMAD2 protein levels were analyzed by Western blot as a positive control for TGFβ efficiency. Both MCEMP1 and Phospho-SMAD2 protein levels were noted to be increased at 12 and 24 h (*D*). The observation that TGFβ increased MCMEP1 was confirmed and by live cell immunostaining (*E*). One-way ANOVA, ****P < 0.0001, ***P < 0.001. Scale bar = 200 μm. MCEMP1, mast-cell expressed membrane protein-1; TGFβ, transforming growth factor beta.

Once we confirmed classical monocytes as one of the predominant *MCEMP1*-expressing cells in IPF, we used the monocytic cell line THP-1 as an in vitro model to elucidate the molecular mechanisms regulating the expression of this gene. THP-1 cells were treated with several compounds known to promote inflammation and/or fibrosis: tumor necrosis factor-α (TNFα, 10 ng/mL), fibroblast growth factor-2 (FGF2, 100 ng/mL), TGFβ (5 ng/mL), or lipopolysaccharide (LPS, 50 ng/mL). We analyzed *MCEMP1* levels by RT-qPCR and Western blot 24 h posttreatment, and we found that TGFβ increased MCEMP1 both at the mRNA and at the protein levels, whereas TNFα, FGF2, and LPS did not have any effect (Fig. 2, *A* and *B*). THP-1 cells were also stimulated with profibrotic interleukins-4 and -13 and proinflammatory interleukin-10 (10 ng/mL), but no effect on *MCEMP1* expression was seen (Fig. 2*C*). *MCEMP1* overexpression happened as early as 12 h. Phospho-SMAD2 and total SMAD2 protein levels were analyzed by Western blot as a positive control for TGFβ efficiency, and levels were noted to be increased at 12 and 24 h (Fig. 2*D*). The observation that TGFβ induces MCEMP1 expression was confirmed by live cell imaging of THP-1 cells immunostained with an MCEMP1 antibody and with a CD14 antibody as a control (Fig. 2*E*).

### TGFβ-Mediated *MCEMP1* Upregulation Results from the Cooperation of SMAD3 and SP1 via Concomitant Binding to SMAD3/SP1 *Cis*-Regulatory Elements within the MCEMP1 Promoter Region

**Figure 3.**
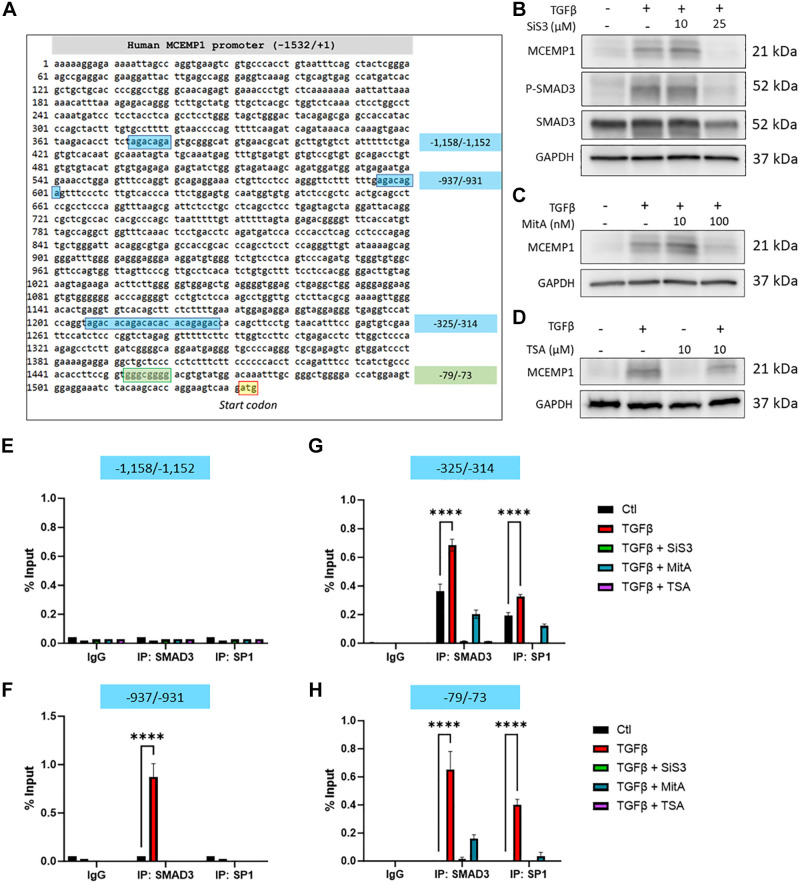
TGFβ-mediated MCEMP1 upregulation results from the cooperation of SMAD3 and SP1 via concomitant binding to SMAD3/SP1 *cis*-regulatory elements within the MCEMP1 promoter region. Analysis of the sequence of a 1.5 kbp promoter region upstream of the human MCEMP1 gene ATG start codon resulted in the identification of three putative SBE/CAGA sites at locations −1,158/−1,152 ( TCT**AGAC**AGA), −937/−931 ( **AGAC**AGA), and −325/−314 ( **AGAC**AC**AGAC**ACACACAG**AGAC**) and a GC box at position −79/73 ( GGGCGGG) (*A*). TGFβ failed to upregulate MCEMP1 expression in the presence of the SMAD3 inhibitor SiS3 (*B*) or SP1 inhibitor mithramycin A (MitA) (*C*). Similarly, THP-1 treatment with trichostatin A (TSA), also blocked MCEMP1 upregulation following TGFβ stimulation (*D*). Chromatin immunoprecipitation followed by qPCR shows that neither SMAD3 nor SP1 can bind to the −1,158/−1,152 SBE/CAGA site (*E*). SMAD3 binds to the −937/−931 SBE/CAGA site, but not SP1 (*F*). Concomitant binding of SMAD3 and SP1 is observed at locations −325/−314 and −79/−73 following THP-1 stimulation with TGFβ. This effect is inhibited in the presence of SiS3 or MitA (*G*, *H*). MCEMP1, mast-cell expressed membrane protein-1; TGFβ, transforming growth factor beta. ****P < 0.0001.

We used the National Institutes of Health (NIH) genetic sequence database GenBank to extract and analyze the sequence of a 1.5 kbp promoter region upstream of the human *MCEMP1* gene ATG start codon to identify SMAD-binding element (SBE) and CAGA boxes (Fig. 3*A*). Our analysis was set up with a minimum sequence homology of 0.7 and a core sequence homology of 1, which resulted in the identification of three putative SBE/CAGA sites at locations −1,158/−1,152 ( TCT**AGAC**AGA), −937/−931 ( **AGAC**AGA), and −325/−314 ( **AGAC**AC**AGAC**ACACACAG**AGAC**). The proximal part of the MCEMP1 promoter is a GC-rich region with a GC-box at position −79/−73 ( GGGCGGG; sequence homology 1, core sequence homology 1) (Fig. 3*A*).

To determine if SMAD3 and/or SP1 are transcriptional effectors in the induction of *MCEMP1* expression by TGFβ, THP-1 cells were treated either with SMAD3 inhibitor SiS3 or SP1 inhibitor mithramycin A (MitA), before TGFβ stimulation. Twenty-four hours later, we observed that TGFβ failed to increase *MCEMP1* expression in the presence of either inhibitor (Fig. 3, *B* and *C*). This suggests that TGFβ requires both SMAD3 and SP1 to induce *MCEMP1* expression. Similarly, THP-1 treatment with trichostatin A (TSA), a histone deacetylase (HDAC) inhibitor known to block SP1 function, also blocked *MCEMP1* upregulation following TGFβ stimulation, confirming the pivotal role of SP1 in this process (Fig. 3*D*) (16).

To assess SMAD3 and SP1 binding to the *MCEMP1* promoter in THP-1 cells, we performed ChiP to scan the regions spanning the *cis*-regulatory elements identified in silico. qPCR amplification following SMAD3 and SP1 immunoprecipitation revealed that SMAD3, but not SP1, is recruited at site −937/−931 following TGFβ stimulation (Fig. 3*F*). This effect was abrogated in the presence of SiS3. Both SMAD3 and SP1 were found to bind to site −325/−314 and to the GC-box (−79/−73), but not in the presence of SiS3, MitA, and TSA (Fig. 3, *G* and *H*). Neither SMAD3 nor SP1 binding was detected on the predicted −1,158/−1,152 SBE/CAGA site (Fig. 3*E*). Taken together, our results demonstrate that TGFβ-mediated MCEMP1 overexpression results from the cooperation of SMAD3 and SP1 via concomitant binding to SMAD3/SP1 *cis*-regulatory elements within the *MCEMP1* promoter.

### Proximity Ligation Assay Identifies the Interaction of SMAD3 and SP1 In Situ, Which Is Enhanced by TGFβ Treatment

**Figure 4.**
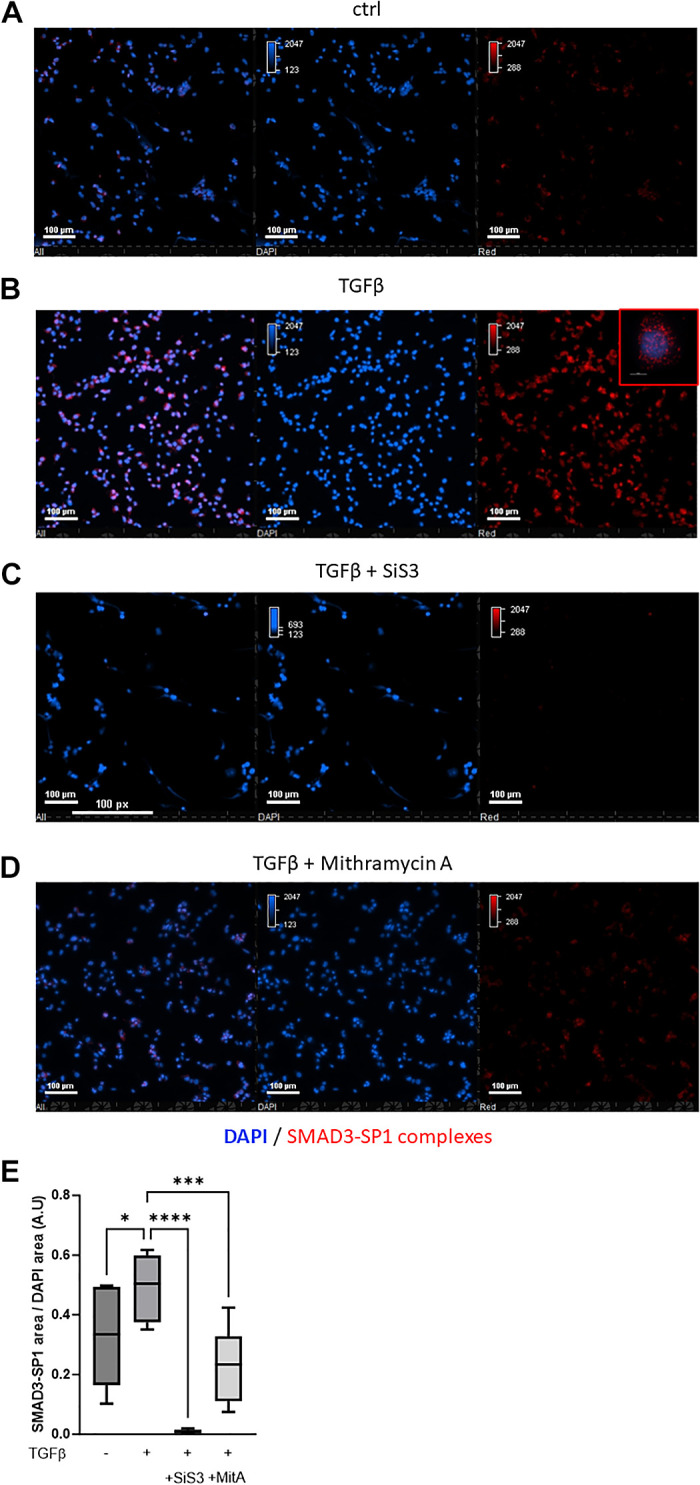
Proximity ligation assay identifies the interaction of SMAD3 and SP1 in situ, which is enhanced by TGFβ treatment. TGFβ treatment resulted in an important increase of fluorescent spots following TGFβ treatment compared with control, particularly in nuclei where both transcription factors translocate to regulate gene expression (*A*, *B*, and *E*). Both SiS3 and MitA drastically reduced the formation of SMAD3-SP1 complexes in THP-1 nuclei, demonstrating the specificity of the PLA signal (*C*, *D*, and *E*). DAPI (Blue), SMAD3-SP1 complexes (Red). ****P < 0.0001, ***P < 0.001, *P < 0.05. DAPI, 4′,6-diamidino-2-phenylindole; TGFβ, transforming growth factor beta.

To visualize SMAD3 and SP1 interaction in THP-1 cells, we performed a proximity ligation assay (PLA) in THP-1 cells cultured and treated with TGFβ in the presence or absence of SiS3 or mithramycin A. We used primary antibodies targeting each protein first, and secondary antibodies labeled with oligonucleotide probes. In our experiment, we observed an important increase of fluorescent spots following TGFβ treatment compared with control, particularly in nuclei where both transcription factors translocate to regulate gene expression (Fig. 4, *A*, *B*, and *E*). Both SiS3 and MitA drastically reduced the formation of SMAD3-SP1 complexes in THP-1 nuclei, demonstrating the specificity of the PLA signal (Fig. 4, *C*–*E*).

### *MCEMP1* Regulates TGFβ-Mediated Monocyte Chemotaxis, Adhesion, and Migration

**Figure 5.**
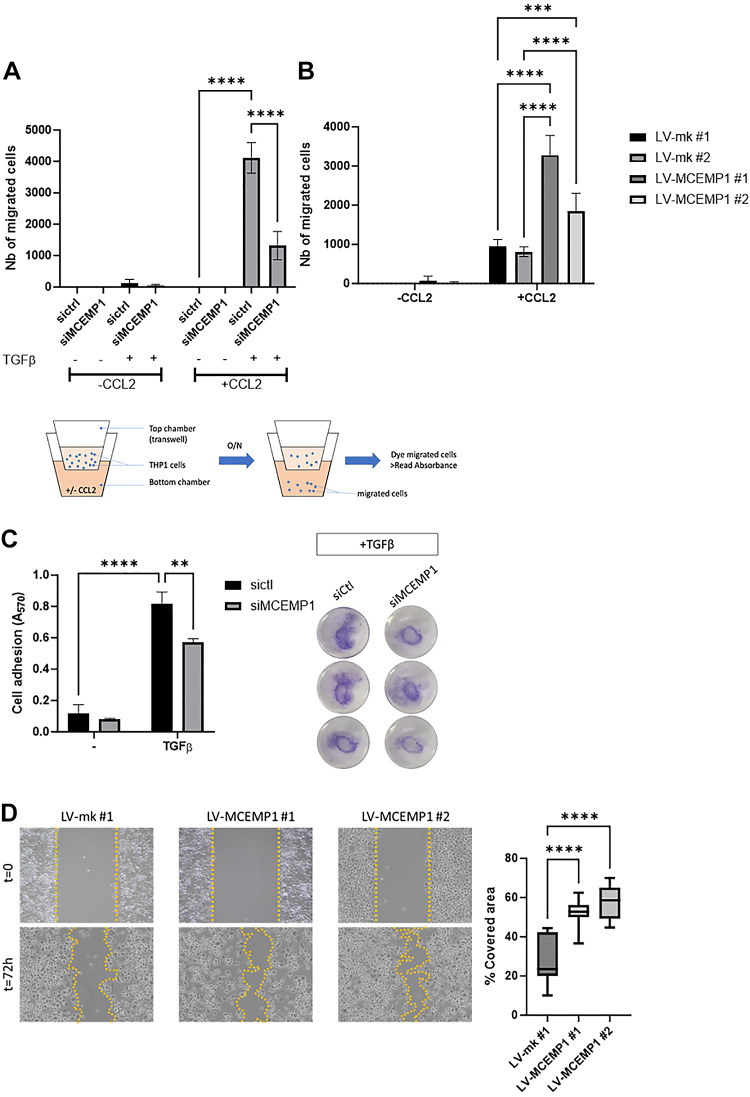
*MCEMP1* regulates TGFβ-mediated monocyte chemotaxis, adhesion, and migration. THP1 cells treated with TGFβ and transfected with *MCEMP1*-targeting siRNA migrate significantly less after CCL2 stimulation (*A*). THP1 cells stably transduced with *MCEMP1*-encoding lentiviral vectors (LV-*MCEMP1*) showed increased migration when compared with cells transduced with an empty vector. Two different clones were tested (*B*). Although TGFβ treatment potently increased THP-1 attachment to the cell culture dish, this effect was partially impaired by *MCEMP1* knockdown (*C*). Scratch assay results show that *MCEMP1* overexpression increases macrophage migration capacity compared with control cells (*D*). Two-way ANOVA, ****P < 0.0001, ***P < 0.001, **P < 0.01. ANOVA, analysis of variance; *MCEMP1*, mast-cell expressed membrane protein-1; TGFβ, transforming growth factor beta.

Once we determined that *MCEMP1* expression was detected in classical monocytes and alveolar macrophages in IPF, and regulated by TGFβ signaling, we then hypothesized that MCEMP1 could participate in the transition of circulating monocytes to monocyte-derived alveolar macrophages by controlling cell chemotaxis and/or migration in a TGFβ-rich environment. To test our hypothesis, we performed a cell migration/chemotaxis assay in THP-1 cells with either increased or decreased expression of *MCEMP1* using Transwell and CCL2 (MCP-1) as a chemoattractant. The efficiency of transfection and transduction for *MCEMP1* knockdown and knock-in was confirmed by Western blot (Supplemental Fig. S1, *A–D*). As shown in Fig. 5*A*, THP-1 cells did not cross the Transwell membrane in the absence of CCL2. In the presence of CCL2, we observed a dramatic increase in Transwell migration upon TGFβ stimulation. This effect was reduced by ∼75% following *MCEMP1* knockdown. To determine whether *MCEMP1* overexpression could increase THP-1 chemotaxis and migration in the absence of TGFβ, we stably transduced THP-1 cells with mock- and *MCEMP1*-lentiviral vectors to induce *MCEMP1* expression. We then performed a chemotaxis/migration assay using the same system as in Fig. 5*A* and tested cells from two different transductions as biological replicates, both for mock- and *MCEMP1*-lentivirus. We observed that *MCEMP1*-overexpressing cells from both transductions migrated more than mock control cells, which indicates that *MCEMP1* enhances THP-1 chemotaxis (Fig. 5*B*).

Since TGFβ promotes monocyte adhesion, we tested whether *MCEMP1* could be involved in this process by performing a cell adhesion assay using control- or *MCEMP1*-siRNA-transfected THP-1 cells, stimulated or not with TGFβ (17). As expected, TGFβ treatment potently increased THP-1 attachment to the cell culture dish. However, this effect was partially impaired by *MCEMP1* knockdown, which suggests a role for *MCEMP1* in TGFβ-mediated monocyte adhesion (Fig. 5*C*). The overexpression of *MCEMP1* without addition of TGFβ failed to induce THP-1 adhesion (data not shown). These observations demonstrate that *MCEMP1* partially controls TGFβ-mediated monocyte adhesion in THP-1 cells.

Next, we aimed to check if *MCEMP1* could control THP-1 migration following differentiation into macrophages. We seeded mock- and *MCEMP1*-overexpressing THP-1 in six-well culture plates and treated them with PMA to induce cell differentiation. The results of a scratch assay showed that *MCEMP1* overexpression increases macrophage migration capacity compared with control cells (Fig. 5*D*). Altogether, our experiments provide evidence that *MCEMP1* regulates monocyte and macrophage chemotaxis, adhesion, and migration, upon exposure to TGFβ.

### *MCEMP1* Mediates RHO-GTPase Activity following TGFβ Stimulation

**Figure 6.**
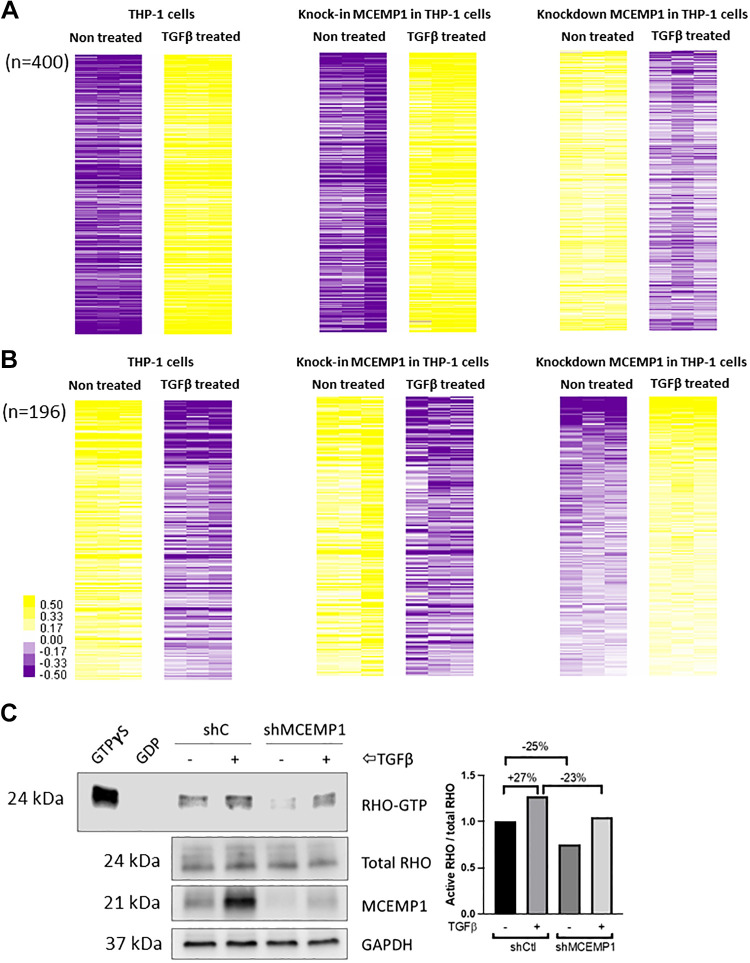
*MCEMP1* mediates Rho-GTPase activity following TGFβ stimulation. Heatmap depicts differentially expressed genes (DEGs) from bulk RNA sequencing of three groups of THP1 cells. *n* = 400 DEGs were found to increase after TGFβ stimulation, further increased in *MCEMP1* overexpressing cells treated with TGFβ and decreased in sh*MCEMP1* cells treated with TGFβ (*A*). *n* = 196 genes followed the opposite pattern of expression (*B*). Color scale is shown adjacent to heat map in log_2_ scale; generally, yellow denotes increase over the geometric mean of samples, and purple, decrease. Differential gene expression analysis based on the negative binomial distribution. FDR < 5%. THP1 cells transfected with *MCEMP1*-targeting shRNA (shM) and treated with TGFβ have a 23% reduction in active Rho-GTPase activity compared with sh control cells treated with TGFβ (*C*). *MCEMP1*, mast-cell expressed membrane protein-1; TGFβ, transforming growth factor beta.

To study the mechanisms by which *MCEMP1* participates in monocyte motility, we performed bulk RNA sequencing in three groups of THP1 cells: nontreated versus TGFβ treated cells, *MCEMP1* mock versus *MCEMP1* overexpressing cells treated with TGFβ (knock-in *MCEMP1*), and sh-control versus sh*MCEMP1* cells treated with TGFβ (knockdown *MCEMP1*). Differentially expressed genes (DEG) with *P* value <0.05 (FDR adjusted) were selected for downstream analyses. We identified *n* = 400 DEG that increased after TGFβ stimulation, further increased in *MCEMP1*-overexpressing cells treated with TGFβ and decreased in sh*MCEMP1* cells treated with TGFβ (Fig. 6*A*). We also identified *n* = 196 genes with the opposite pattern of expression (Fig. 6*B*). For enrichment analyses, we focused on the former groups of DEG genes (Fig. 6*A*). GO analysis of biological processes demonstrated enrichment for positive regulation of GTPase activity and signal transduction (Table 2).

**Table 2.** GO annotation analysis of n = 400 differentially expressed genes responsive to TGFβ and regulated by MCEMP1 in THP-1 cells show enrichment for positive regulation of GTPase activity and signal transduction

GO Biological Process	Genes Expected	Genes Identified	Fold Change	*P* Value
Establishment of epithelial cell polarity (GO:0090162)	35	8	11.71	1.26E-02
Transforming growth factor β receptor signaling pathway (GO:0007179)	96	13	6.94	1.58E-03
Cellular response to transforming growth fact β stimulus (GO:0071560)	155	15	4.96	9.57E-03
Response to transforming growth factor β (GO:0071559)	162	15	4.74	1.59E-2
Regulation of small GPTase-mediated signal transduction (GO:0051056)	304	28	4.72	5.71E-07
Bone development (GO:0060348)	220	19	4.42	1.82E-03
Positive regulation of GTPase activity (GO:0043547)	278	22	4.05	8.26E-04
Response to hypoxia (GO:0001666)	274	20	3.74	1.04E-02
Endocytosis (GO:0006897)	534	38	3.64	2.78E-07
Regulation of GPTase activity (GO:0043087)	368	26	3.62	4.69E-04

Bonferroni corrected *P* value <0.05. GO, gene ontology; MCEMP1, mast-cell expressed membrane protein-1.

RHO GTPases such as RHO, RAC, and CDC42 have been shown to play an essential role in the regulation of monocyte/macrophage chemotaxis and migration in response to chemokines such as CCL2 or colony-stimulating factor-1 (CSF-1) (18). Therefore, we monitored RHO activation following TGFβ stimulation in THP-1 stably transduced with control- or *MCEMP1*-shRNA. We found that TGFβ enhances RHO GTPase activity; however, this effect was attenuated following *MCEMP1* knockdown (Fig. 6*C*). Taken together, these results support the role of *MCEMP1* in TGFβ-mediated motility of monocytes through the modulation of RHO activity.

## DISCUSSION

Our study provides both human data and strong in vitro evidence for the role of *MCEMP1* in the pathogenesis of pulmonary fibrosis. We showed through single-cell RNA sequencing that *MCEMP1* is expressed primarily in classical monocytes and alveolar macrophages in IPF. Thus, we used a monocytic cell line (THP-1 cells) for our mechanistic studies and demonstrated that MCEMP1 is a transcriptional target of TGFβ, a key mediator of pulmonary fibrosis. TGFβ transduces signals through canonical and noncanonical pathways (19–21). The well-characterized canonical TGFβ signaling pathway uses SMAD2 and/or SMAD3 along with SMAD4 to transfer signals, and SMAD3 and SMAD4 are able to directly bind to DNA through two different consensus sequences: GTCTAGAC, called SMAD binding element or SBE; and AG(C/A) CAGACAC, called CAGA box. Both sequences contain the core motif **AGAC**, which represents the optimal binding sequence for SMAD3 and SMAD4 (22, 23). Here we show that TGFβ signaling regulates MCEMP1 expression in a noncanonical fashion by enhancing SMAD3 and SP1 interaction and concomitant binding to SMAD3/SP1 *cis*-regulatory elements in positions −325/−314 and −79/−73 within the MCEMP1 promoter. These results corroborate previous studies describing SMAD3-SP1 transcriptional complexes (24, 25). In addition, our study provides evidence that MCEMP1 enhances TGFβ-mediated monocyte adhesion, chemotaxis, and migration by modulating the effect of TGFβ in RHO activity.

Once we determined that MCEMP1 expression is regulated by TGFβ-SMAD3-SP1 and mainly detected in monocytes, we sought to understand the function of the protein. Monocytes are cells with migratory capacity, being in the circulation, but they also leave this compartment and migrate to peripheral tissues where they differentiate into macrophages (26). We thus speculated and showed that MCEMP1 is involved in monocyte trafficking by controlling chemotaxis and/or migration. Given that TGFβ promotes monocyte migration and adhesion as well as macrophage migration through RHO GTPases, we tested whether MCEMP1 could be involved in this process (18, 27–30). Our results provide evidence that increased *MCEMP1* expression following monocyte and macrophage exposure to TGFβ enhances cell chemotaxis, adhesion, and migration by regulating the effect of TGFβ in RHO GTPase signaling. Finally, we identified the presence of MCEMP1 in lung tissue monocytes and macrophages, particularly in alveolar macrophages in IPF, suggesting that these cells could be monocyte-derived and that MCEMP1 may participate in the migration and transition of monocytes to monocyte-derived alveolar macrophages. Furthermore, in vivo studies will be required to demonstrate that alveolar macrophages in IPF expressing MCEMP1 are monocyte-derived.

Our findings showing a role of *MCEMP1* in THP-1 cell chemotaxis and migration is in line with mechanistic data of MCEMP1 function in the context of other diseases (31, 32). In particular, it has been demonstrated that MCEMP1 may affect the proliferation, migration, and invasion of gastric cancer cells by regulating epithelial-to-mesenchymal transition (32). It is also known that small GTPases of the RHO/Rac family participate in TGFβ-induced EMT and cell motility in cancer (30), an effect that based on our results seems to be mediated in monocytes and macrophages in IPF, at least in part by MCEMP1. In addition to pulmonary fibrosis, a recent study demonstrated that MCEMP1 deficiency blunted SCF-induced peritoneal mast cell proliferation in vitro and lung mast cell expansion in vivo*. Mcemp1*-deficient mice had decreased airway inflammation and lung impairment in mouse models of chronic asthma (33). These findings highlight that MCEMP1 might be an emerging key mediator for multiple diseases. In addition, elevated MCEMP1 has been recently reported as a negative prognostic marker in COVID-19 as well as other diseases such as stroke, thus is possible that MCEMP1 participates in fibrosis post injury (34–36).

Our study has some limitations. First, we did not use primary human monocytes for our mechanistic experiments, but a monocytic cell line (THP-1 cells). Primary monocytes are short lived and difficult to transfect or transduce. We used THP-1 cells because MCEMP1 was first described in THP-1 cells (11). THP-1 cells exhibit a fast growth rate, are homogenous in cell culture, and can differentiate. Also, THP-1 cells are a widely used and validated model to study monocyte biology (37). Second, we did not validate our results in vivo; yet we provide strong in vitro evidence for the role of MCEMP1 in pulmonary fibrosis.

Taken together, our study has several important attributes. Our data demonstrate that *MCEMP1*, a gene of a 52-gene, high-risk profile predictive of IPF mortality when upregulated, is highly expressed in classical monocytes in PBMC and alveolar macrophages in IPF lung tissues when compared with control lungs. Regarding the cellular source of MCEMP1, our findings suggest that its name might be a misnomer, given that MCEMP1 is not exclusively expressed in mast cells. Our report is also the first study providing mechanistic insights for the role of MCEMP1 in pulmonary fibrosis and one of the few studies identifying the mechanistic role of MCEMP1 in general. Our study not only identified that MCEMP1 expression is regulated by the TGFβ-SMAD3-SP1 axis but also that MCEMP1 seems to be involved in the cross talk between TGFβ and RHO-GTPase in monocyte chemotaxis and macrophage migration, a mechanism that has not been previously reported. The findings noted above imply a potentially important mechanistic role of MCEMP1 for pulmonary fibrosis development and progression. Mcemp1 knock-in and knockout mouse models will be required in future studies to further validate our in vitro findings.

## ETHICS APPROVAL

Protocol, data collection and analysis were approved by the Institutional Review Board and the Local Ethics Committee (BullsIRB protocol: Pro00032158).

## DATA AVAILABILITY

Data will be made available upon reasonable request.

## SUPPLEMENTAL DATA

10.6084/m9.figshare.24878472 Supplemental Table S1 and Fig. S1: https://doi.org/10.6084/m9.figshare.24878472.

## GRANTS

This study was funded by the National Institutes of Health Grants F30HL162459, R01HL127349, R01HL141852, U01HL145567, R21HL161723, P01HL11450, and a grant from the Three Lakes Foundation (to N.K.), Pulmonary Fibrosis Foundation Scholars Award (to A.U.) and Ubben Center for Pulmonary Fibrosis Research Fund (to J.D.H.-M.).

## DISCLOSURES

N. Kaminski is a scientific founder at Thyron, served as a consultant to Biogen Idec, Boehringer Ingelheim, Third Rock, Pliant, Samumed, NuMedii, Theravance, LifeMax, Three Lake Partners, Optikira, Astra Zeneca, RohBar, Veracyte, Augmanity, CSL Behring, Galapagos, Fibrogen, and Thyron over the past 3 years, reports Equity in Pliant and Thyron, and grants from Veracyte, Boehringer Ingelheim, BMS and nonfinancial support from MiRagen and Astra Zeneca. A. Unterman reports receiving research funding from Boehringer Ingelheim, and personal consulting fees or honoraria from Boehringer Ingelheim, Kamada, RemedyCell, Augmanity Nano, Splisense, Veracyte, and 1E Therapeutics. None of the other authors has any conflicts of interest, financial or otherwise, to disclose.

## AUTHOR CONTRIBUTIONS

C.Y.P., T.K., N.K., and J.D.H.-M. conceived and designed research; C.Y.P., T.K., A.U., T.A., K.M., A.A., A.Z., N.K., G.K., K.P., D.B., and J.D.H.-M. performed experiments; C.Y.P., T.K., A.U., T.A., K.M., A.A., A.Z., N.K., G.K., K.P., D.B., and J.D.H.-M. analyzed data; C.Y.P., T.K., A.U., T.A., K.M., A.A., A.Z., N.K., G.K., K.P., D.B., and J.D.H.-M. interpreted results of experiments; C.Y.P., T.K., A.U., T.A., K.M., A.A., A.Z., N.K., D.B., and J.D.H.-M. prepared figures; C.Y.P., T.K., N.K., and J.D.H.-M. drafted manuscript; C.Y.P., T.K., A.U., T.A., K.M., A.A., A.Z., N.K., G.K., K.P., D.B., and J.D.H.-M. edited and revised manuscript; C.Y.P., T.K., A.U., T.A., K.M., A.A., A.Z., N.K., G.K., K.P., D.B., and J.D.H.-M. approved final version of manuscript.
